# N-Substituted Pyrrole Derivative 12m Inhibits HIV-1 Entry by Targeting Gp41 of HIV-1 Envelope Glycoprotein

**DOI:** 10.3389/fphar.2019.00859

**Published:** 2019-08-02

**Authors:** Jiayin Qiu, Taizhen Liang, Junyan Wu, Fei Yu, Xiaoyang He, Yuanxin Tian, Lan Xie, Shibo Jiang, Shuwen Liu, Lin Li

**Affiliations:** ^1^Guangdong Provincial Key Laboratory of New Drug Screening, Guangzhou Key Laboratory of Drug Research for Emerging Virus Prevention and Treatment, School of Pharmaceutical Sciences, Southern Medical University, Guangzhou, China; ^2^School of Pharmaceutical Science, Zhejiang Chinese Medical University, Hangzhou, China; ^3^Department of Pharmacy, Sun Yat-sen Memorial Hospital, Sun Yat-sen University, Guangzhou, China; ^4^College of Life Sciences, Agricultural University of Hebei, Baoding, China; ^5^Key Laboratory of Medical Molecular Virology of Ministries of Education and Health, Shanghai Medical College, Fudan University, Shanghai, China; ^6^Beijing Institute of Pharmacology and Toxicology, Beijing, China

**Keywords:** HIV-1 entry inhibitor, gp41 envelope, six-helix bundle, cell–cell fusion, small molecular compound

## Abstract

The combination of three or more antiviral agents that act on different targets is known as highly active antiretroviral therapy (HAART), which is widely used to control HIV infection. However, because drug resistance and adverse effects occur after long-term administration, an increasing number of HIV/AIDS patients do not tolerate HAART. It is necessary to continue developing novel anti-HIV drugs, particularly HIV entry/fusion inhibitors. Our group previously identified a small-molecule compound, NB-64, with weak anti-HIV activity. Here, we found that N-substituted pyrrole derivative 12m (NSPD-12m), which was derived from NB-64, had strong anti-HIV-1 activity, and NSPD-12m-treated cells showed good viability. The mechanism of action of NSPD-12m might be targeting the gp41 transmembrane subunit of the HIV envelope glycoprotein, thus inhibiting HIV entry. Site-directed mutagenesis confirmed that a positively charged lysine residue (K574) located in the gp41 pocket region is pivotal for the binding of NSPD-12m to gp41. These findings suggest that NSPD-12m can serve as a lead compound to develop novel virus entry inhibitors.

## Introduction

Acquired immune deficiency syndrome (AIDS) is caused by the human immunodeficiency virus (HIV) and has become one of the most dangerous epidemics. Currently, treatment with highly active antiretroviral therapy (HAART) combined by three or more different anti-HIV drugs is widely used to reduce the amount of active virus and AIDS-related deaths (McArthur et al., 2003). However, because drug resistance and adverse effects occur during long-term administration, an increasing number of patients do not tolerate HAART, and the drugs are expensive. It is necessary to continue developing novel anti-HIV drugs, particularly HIV entry/fusion inhibitors.

HIV entry into host cells is mediated by HIV-1 envelope glycoprotein (Env). The transmembrane subunit gp41 is critical for the fusion process between the virus and cells (Zhu et al., 2006). Because gp41 contains a relatively conserved amino acid sequence, gp41 is considered one of the most attractive targets for developing HIV entry/fusion inhibitors and preventing drug resistance (Lu et al., 2016; Yi et al., 2016). During the HIV entry process, three C-terminal heptad repeats (CHRs) fold back on three inner N-terminal heptad repeats (NHRs) to form a stable six-helix bundle (6-HB) core structure, bringing the viral and host cell membranes into sufficient proximity for fusion (Jiang et al., 1993). A peptide derived from the CHR region, T20 (Fuzeon), was the first HIV entry/fusion inhibitor by interacting with the gp41 fusion intermediate (Munoz-Barroso et al., 1998). Due to the low oral bioavailability, short half-life, and high production cost of the peptide drug T20, the development of effective, safe, and affordable small-molecule HIV entry/fusion inhibitors is urgently needed. The binding partner of T20, the gp41’s NHR trimer, may serve as an effective target (Liu et al., 2007).

**Figure 1:**
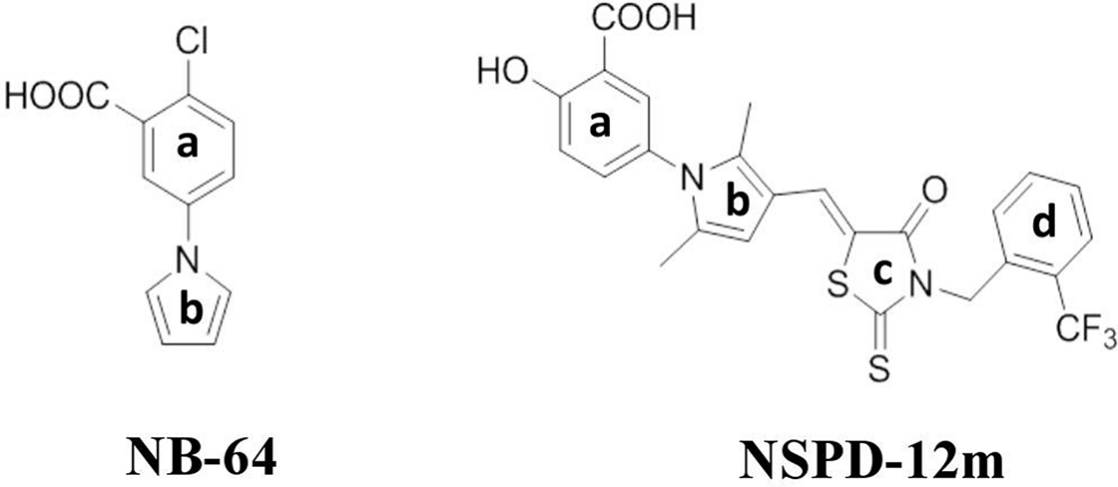
Chemical structures of NB-64 and NSPD-12m.

Our previous studies demonstrated that two “drug-like” small-molecule compounds, NB-2 and NB-64, could inhibit HIV-1 entry by binding to the highly conserved and deep hydrophobic pocket on the surface of gp41’s inner NHR trimer (Jiang et al., 2004). The effective concentration for 50% inhibition (EC_50_) values of NB-2 and NB-64 are on the µM level, which is much higher than the EC_50_ value of T20. Although NB-2 and NB-64 cannot be further developed to bring to market, they are good hit compounds for designing and developing new and more potent inhibitors that target the gp41 binding site. By considering the chemical structure of NB-64 (**Figure 1**), our group further screened a series of small-molecule compounds that target gp41; these compounds exhibited more potent anti-HIV activities than NB-64. In our recent studies, we reported a novel small-molecule compound named N-substituted pyrrole derivative 12m (NSPD-12m) (**Figure 1**) that had improved anti-HIV-1 activity targeting gp41. The chemical name of NSPD-12m is (Z)-N-(3-carboxy-4-hydroxy)phenyl-2,5-dimethyl-(3-(5-(3-(2-(trifluoromethyl)benzyl)-4-oxo-2-thioxothiazolidinylidene)methyl)pyrrole. Here, an N-aryl rhodanine moiety was introduced at the 3-position of N-(3-carboxy) phenyl-2,5-dimethylpyrrole, and the *o*-position on the D-ring was substituted by trifluoromethyl. However, the detailed mechanism of NSPD-12m is still uncertain. In this study, we further evaluated the effects of NSPD-12m on HIV-1 entry/fusion and identified the key target(s) of drug action. This information could provide a new starting point for further structural modifications.

## Materials and Methods

### Reagents, Cells, and Plasmids

HEK-293T cells were purchased from ATCC (Manassas, VA). U87-CD4-CXCR4 cells, U87-CD4-CCR5 cells, TZM-bl cells, MT-2 cells, CHO-WT cells, T20, AMD3100, the *p*NL4-3E^-^R^-^Luc plasmid, HIV-1, and vesicular stomatitis virus-G (VSV-G) Env-encoding plasmids were obtained from the National Institutes of Health AIDS Research and Reference Reagent Program. The peptides N36 and C34 were synthesized by a standard solid-phase Fmoc method by GL Biochem of China and were purified by HPLC. ADS-J1 was purchased from ComGenex (Budapest, Hungary). Mouse mAb NC-1 specific for the gp41 6-HB was prepared and characterized as previously described (Jiang et al., 1998). Horseradish peroxidase (HRP)-labeled goat anti-mouse-IgG and goat anti-rabbit-IgG were purchased from Invitrogen (San Francisco, CA). Polyethyleneimine (PEI), biotin-labeled goat anti-mouse IgG, 2,3-bis (2-methoxy-4-nitro-5-sulfophenyl)-5-(phenylamino) carbonyl-2H-tetrazolium hydroxide [XTT], diethylaminoethyl (DEAE)-dextran and 3,3′,5,5′-tetramethylbenzidine (TMB) were purchased from Sigma (St. Louis, MO). Streptavidin-labeled horseradish peroxidase (SA-HRP) was purchased from Zymed (South San Francisco, CA).

### Synthesis

NSPD-12m was synthesized using the Knoevenagel condensation reactions as previously described (He et al., 2013). Briefly, a mixture of an aldehyde compound N-(3-carboxy-4-hydroxy)phenyl-2,5-dimethyl-3-formylpyrrole and a rhodanine derivative 3-(2-(trifluoromethyl)benzyl)-2-thioxothiazolidin-4-one (mole ratio 1:1) in the presence of ammonium acetate (100 mg) in toluene (20 ml/mM) and methanol (10 ml/mM) was heated to reflux for 3–5 h. After the reaction was finished, ethyl acetate was added to the solution. Water and brine were used to wash the organic phase, successively. After drying over anhydrous sodium sulfate and removing the solvent under reduced pressure, the residue was purified by a flash silica column [gradual eluent: ethyl acetate/petroleum ether with acetic acid (3%), 0–60%] to yield the pure product.

### Anti-HIV-1 Activity

The HIV-1 replication-inhibiting activity of compounds was determined as previously described (Li et al., 2013). Briefly, 1 × 10^4^ TZM-bl or MT-2 cells were infected with primary HIV-1 strains or T20-resistant HIV-1 strains (100 TCID_50_) in 200 µl of RPMI-1640 medium containing 10% FBS in the presence or absence of compounds at graded concentrations for 12 h. The culture medium was replaced with fresh medium without the compounds. After culture for an additional 48 h, supernatants or cells were collected from each well. For the primary HIV-1 strains, the cells were treated using a luciferase kit. Briefly, the cells were lysed with the lysis reagent and were transferred into a 96-well flat-bottom luminometer plate after three washes with phosphate-buffered saline (PBS) (Costar Corning Inc., Corning, NY), followed by addition of the luciferase substrate included in the luciferase kit (Promega Corp., Madison, WI). The luciferase activity was measured immediately by an Ultra 384 microplate reader (Tecan, Research Triangle Park, NC). T20 was used as a positive control. For the T20-resistant HIV-1 strains, 100 µl of culture supernatant was mixed with 100 µl of 5% Triton X-100. Then, those virus lysates were assayed for p24 antigen by enzyme linked immunosorbent assay (ELISA) as previously described (Li et al., 2010). Absorbance at 450 nm was recorded in an ELISA reader (Ultra 384, Tecan). EC_50_ values were calculated using the computer program CalcuSyn (Chou and Talalay, 1984). T20 and ADS-J1 (a small-molecule HIV-1 entry inhibitor that blocks gp41 6-HB formation) were used as negative controls.

### XTT Assay

The *in vitro* cell viability of NSPD-12m on HIV-1 target cells (U87-CD4-CXCR4, U87-CD4-CCR5, TZM-bl, and MT-2 cells) and HIV effector cells (CHO-WT cells) was analyzed by an XTT colorimetric assay. Briefly, 100 µl of 5 × 10^5^/ml cells was mixed and incubated with 100 µl of NSPD-12m at graded concentrations at 37°C for 3 days. Then, 50 µl of XTT solution (1 mg/ml) containing 0.02 µM of phenazinemethosulphate (PMS) was added to each well, and the cells were incubated at 37°C for another 4 h. The absorbance at 450 nm was measured with an ELISA reader. The 50% cytotoxicity concentrations (CC_50_) were calculated using the CalcuSyn software (Chou and Talalay, 1984).

### Generation of HIV-1 or VSV-G Pseudoviruses

HIV-1 pseudoviruses were prepared as previously described (Yin et al., 2018). Briefly, HEK-293T cells were cotransfected with a *p*NL4-3E^-^R^-^Luc plasmid and different HIV-1 Env-encoding plasmids derived from HIV-1_HXB2_ (X4 strain), HIV-1_JR-FL_ (R5 strain), HIV-1_SF162_ (R5 strain), or VSV-G Env-encoding plasmids by using a PEI transfection reagent. Cell culture supernatants containing HIV-1 or VSV-G pseudoviruses were harvested and centrifuged at 72 h posttransfection and were then stored in a −80ºC refrigerator.

### A Single-Cycle Infection Assay

The inhibitory activity of NSPD-12m against infection by different HIV-1 or VSV-G pseudotyped viruses was assessed as described previously (Yin et al., 2018). Briefly, 100 µl of U87-CD4-CXCR4 or U87-CD4-CCR5 cells were seeded at 1 × 10^4^ cells/well in a 96-well tissue culture plate at 37°C overnight. In addition, then, HIV-1_HXB2_, HIV-1_JR-FL_, HIV-1_SF162_, or VSV-G pseudoviruses containing DEAE-dextran (final concentration of 15 μg/ml) were added to the previously discussed cells. At 24-h postinfection, the culture supernatants were discarded, and fresh media were added and further incubated for 48 h. The luciferase activity was measured immediately as described previously. T20 or AMD3100 was used as a positive control.

### HIV-1 Env-Mediated Cell–Cell Fusion

Here, a noninfectious cell–cell fusion assay was used to determine the effect of NSPD-12m on the early step of HIV-1 entry into a target cell as described previously (Li et al., 2010). Briefly, 1 × 10^5^ CHO-WT cells expressing the HIV-1 envelope glycoprotein gp160 (as the effector cells) were preincubated with graded concentrations of NSPD-12m at 37°C for 30 min. Then, 1 × 10^5^ MT-2 cells expressing the CD4 receptor and the CXCR4 coreceptor (as the target cells) were added and cocultured for another 48 h. The obvious syncytia could be counted under an inverted microscope (Nikon, Japan). Four fields per well were examined randomly. The EC_50_ values were calculated as described previously (Chou and Talalay, 1984).

Inhibition of fusion between MT-2 cells infected by HIV-1 strains (X4 and R5 viruses) with CEMx174 5.25 M7 cells, which express CD4 and both coreceptors, CXCR4 and CCR5, was determined by a luciferase assay. Briefly, MT-2 cells were infected with the corresponding HIV-1 strains for 7 days. The infected MT-2 cells were washed three times and resuspended in culture medium to 2 × 10^5^/ml. Then, 50 μl of a compound at graded concentrations in triplicate was incubated with equal volumes of HIV-1-infected MT-2 cells at 37°C for 30 min. Next, 100 μl of CEMx174 5.25 M7 cells (4 × 10^5^/ml) was added, and the cells were incubated at 37°C for 3 days. The cells were collected, washed, and lysed with the lysing reagent included in the luciferase kit (Promega, Corp., Madison, Wis). Aliquots of cell lysates were transferred to 96-well flat-bottom luminometer plates (Costar), followed by the addition of luciferase substrate (Promega). Luciferase activity was measured using an Ultra 384 luminometer. ADS-J1 was used as a positive control for all cell–cell fusion assays.

### A Sandwich ELISA

The inhibition of gp41 6-HB formation by NSPD-12m was determined by a sandwich ELISA as previously described (Liu et al., 2007). In brief, the wells of a 96-well polystyrene plate (Corning) were first precoated with 2 µg/ml IgG purified from rabbit antisera against the N36/C34 complex at 4°C overnight. In addition, the wells were blocked by 2% dry nonfat milk at 37°C for 1 h. NSPD-12m at graded concentrations was incubated with 0.5-µM N36 at 37°C for 30 min, followed by the addition of 0.5-µM C34 at 37°C for another 30-min incubation. Afterward, the previously mentioned mixtures were added to the prepared wells and incubated at 37°C for another 1 h. Then, 1 µg/ml mAb NC-1, biotin-labeled goat anti-mouse IgG (1:10,000 diluted in PBS buffer with 2% dry nonfat milk), SA-HRP (1:20,000 diluted in PBS buffer with 10% goat serum), TMB, and 1-M H_2_SO_4_ were added sequentially. A450 was measured by an ELISA reader, and EC_50_ was calculated using CalcuSyn software (Chou and Talalay, 1984). NB-64, a small-molecule HIV entry inhibitor targeting gp41, and ADS-J1 were used as positive controls.

### Native Polyacrylamide Gel Electrophoresis (N-PAGE)

The inhibition of gp41 6-HB formation by NSPD-12m was further confirmed by N-PAGE as reported previously (Liu et al., 2005). Briefly, NSPD-12m at the indicated concentrations was incubated with the peptide N36 (100 μM in PBS) at 37°C for 30 min before the addition of the peptide C34 (100 μM in PBS). After incubation at 37°C for another 30 min, the mixture was diluted in Tris-glycine native sample buffer (Invitrogen, Carlsbad, CA) and then loaded onto 10 × 1.0-cm precast Tris-glycine gels (18%, Invitrogen) at 20 µl/well. Gel electrophoresis was carried out at constant voltage of 120 V at room temperature (RT) for 2 h in Tris-glycine native running buffer. Immediately after electrophoresis, the gel was stained by Coomassie blue (Invitrogen) and imaged by the FluorChem 8800 Imaging System (Alpha Innotech, San Leandro, CA). ADS-J1 (1 mM in PBS), a small-molecule HIV-1 entry inhibitor that blocks gp41 6-HB formation, was used as a positive control (Wang et al., 2009).

### Circular Dichroism (CD) Spectroscopy

The effects of NSPD-12m on the formation of the secondary α-helix structures of the peptides N36 and C34 were detected by CD spectroscopy as described previously (Liu et al., 2005; Zhang et al., 2018). Briefly, the peptide N36 (10 µM in PBS) was incubated with NSPD-12m (20 µM in PBS) or PBS at 37°C for 30 min, followed by incubation with the peptide C34 (10 µM in PBS) at 37°C for another 30 min. To further confirm that NSPD-12m inhibited the formation of secondary α-helix structures, the peptide N36 was incubated with the peptide C34 at 37°C for 30 min before the addition of the compound. ADS-J1 (40 µM in PBS) was used as a positive control. The reported CD spectra of the peptides N36 and C34 and the mixture of N36, C34, and the compounds described previously were obtained at 25°C using a Jasco 715 spectropolarimeter (Jasco Inc., Japan) with a 5.0-nm bandwidth, 0.1-nm resolution, 0.1-cm path length, 4.0-s response time, and 50-nm/min scanning speed. The spectra were corrected by the subtraction of a blank corresponding to the solvent. The quantities of compounds with α-helix structures were estimated from the molar ellipticity at 222 nm using Jasco software utilities.

### Surface Plasmon Resonance (SPR)

The interaction between NSPD-12m and the synthetic peptide N36 was analyzed by a PlexArray HT system (Plexera Bioscience, WA) as described before (Yin et al., 2018). Briefly, NSPD-12m (10 mM) was immobilized on a chip surface by photo cross-linking. N36 at different concentrations (125, 250, and 500 µM in PBS) was injected at a flow rate of 2 µl/s with an association and dissociation time of 300 s at 25°C. At the end of each cycle, glycine–HCl buffer (pH 2.0) at a flow rate of 3 µl/s was used to regenerate the surface of the sensor chip platform with a regeneration time of 300 s. The binding affinity (KD) was calculated using the PlexeraDE data analysis module and GraphPad Prism 5.0 software.

### Molecular Modeling Assay

The AutoDock 4.2 software package was used for flexible docking of the ligand. The macromolecule for docking was downloaded from the RCSB database (PDBID: 1aik). After extracting the six helixes, the PDBQT file was generated in the AutoDock Tools. The residues K574, Arg579, and Gln577 were selected as the flexible residues. The ligands were constructed using the Sketch Molecule module and optimized by the TRIPOS force field integrated in the sybyl7.3 platform. The energy convergence criterion is 0.001 kcal/mol. AutoGrid was used to generate a grid box (40 × 40 × 50 with a grid spacing of 0.375 Å) for docking. The Lamarckian genetic algorithm (LGA) was chosen to perform the docking with default parameters except that the max conformation per ligand was 100. The docking results were sorted by the binding energy of the most populated cluster using AutoDock Tools, and the highest ranked results were chosen for the interaction analysis.

### Site-Directed Mutagenesis

The plasmid encoding HIV-1 JR-FL-Env was used to generate the wild-type HIV-1_JR-FL_ pseudovirus. The design and synthesis of all primers used to construct HIV-1_JR-FL_ Env mutants were performed by following the manufacturer’s instructions (Stratagene, Cedar Creek, TX). Briefly, a series of plasmids encoding HIV-1 JR-FL-Env mutants (W571A, K574A, Q577A and R579A) were prepared by polymerase chain reaction (PCR) using PrimeSTAR^®^ HS DNA Polymerase (Takara, Japan) and were digested by FastDigest DpnI (Fermentas, Lithuania). After confirmation by DNA sequencing, HIV-1 JR-FL-Env mutants were generated by transformation and amplification using those plasmids. In addition, the culture supernatants were then concentrated by PEG-it^™^ Virus Precipitation Solution (System Biosciences, Mountain View, CA) and stored at -80°C until use. The inhibitory activity of NSPD-12m against infection by different HIV-1_JR-FL_ Env mutants and wild-type pseudotyped viruses was evaluated as described previously.

## Results

### NSPD-12m Exhibited Strong Inhibitory Activities Against Infection by Different Primary and Drug-Resistant HIV-1 Strains

To evaluate the inhibitory activities of NSPD-12m, different primary and T20-resistant HIV-1 strains were used to infect TZM-bl cells or MT-2 cells as previously described in the Materials and Methods. As shown in **Table 1**, NSPD-12m showed highly potent inhibitory activities against all tested representative primary HIV-1 strains, including the X4, R5, and X4R5 strains, with EC_50_ values ranging from 2.03 to 6.85 µM. Currently, the sexual transmission of HIV-1 strains is resistant to the currently used antiretroviral therapeutics, including the first HIV entry/fusion inhibitor, T20. Notably, our results showed that NSPD-12m was effective in inhibiting infection by a series of T20-resistant mutants, with EC_50_ values ranging from 15.74 to 24.66 µM. However, the negative control, T20, had no inhibitory activity against any of the previously mentioned T20-resistant mutants even at a T20 concentration (2.0 µM) more than 10 times its EC_90_ against HIV-1 infection (**Table 1**). Another small-molecule HIV-1 entry inhibitor, ADS-J1, showed similar antiviral activities against HIV infection by different primary and drug-resistant strains. These results confirm that NSPD-12m is a potent antiviral compound.

**Table 1:** Inhibitory activities of NSPD-12m on infection by different primary and T20-resistant HIV-1 strains.

Virus strain	Inhibitory activity (Mean ± SD)^a^					
	NSPD-12m		T20		ADS-J1	
	IC_50_ (µM)	IC_90_ (µM)	IC_50_ (µM)	IC_90_ (µM)	IC_50_ (µM)	IC_90_ (µM)
Primary HIV-1 strains						
92UG029	2.078 ± 0.041	2.478 ± 0.041	0.062 ± 0.015	0.270 ± 0.183	20.57 ± 5.29	32.15 ± 6.71
US4 (GS 007)	2.342 ± 0.353	5.274 ± 2.729	0.059 ± 0.010	1.427 ± 0.443	4.62 ± 0.76	9.90 ± 1.44
93/BR/020	4.911 ± 0.130	6.757 ± 1.561	0.445 ± 0.006	0.313 ± 0.022	12.95 ± 3.49	26.95 ± 4.30
BCF02	1.788 ± 0.238	3.480 ± 1.093	0.050 ± 0.022	0.502 ± 0.338	1.45 ± 0.26	3.06 ± 0.42
T20-resistant HIV-1 strains						
NL4-3_(36G)V38A,N42D_	22.402 ± 2.836	32.497 ± 4.946	0.895 ± 0.310	>2	0.65 ± 0.18	8.20 ± 2.65
NL4-3_D36G_	10.822 ± 1.672	55.367 ± 16.943	0.107 ± 0.020	0.881 ± 0.314	0.94 ± 0.03	2.03 ± 0.20
NL4-3_(36G)V38A_	23.657 ± 5.154	32.523 ± 5.416	0.279 ± 0.082	>2	0.16 ± 0.04	0.40 ± 0.05
NL4-3_(36G)N42T,N43K_	4.231 ± 1.919	29.661 ± 2.282	>2	>2	0.61 ± 0.25	4.24 ± 1.21
NL4-3_(36G)V38E,N42S_	5.261 ± 0.762	19.596 ± 0.552	>2	>2	0.11 ± 0.01	0.33 ± 0.01
NL4-3_(36G)V38A,N42T_	16.264 ± 3.030	33.880 ± 6.141	>2	>2	0.62 ± 0.16	8.39 ± 1.55

a NL4-3_D36G_ is a T20-sensitive strain and is the parent strain used for the generation of T20-resistant mutants. The other strains are T20-resistant strains.

### NSPD-12m-Treated HIV-1 Target Cells Showed Good Viability *In Vitro*

The viability of cells treated with NSPD-12m was evaluated with different HIV-1 target cell lines, including the U87-CD4-CXCR4, U87-CD4-CCR5, TZM-bl, and MT-2 cell lines. The cell viability was also assessed for CHO-WT cells expressing HIV-1 envelope proteins. NSPD-12m had relatively low *in vitro* cytotoxicity to all tested cells, suggesting that NSPD-12m has the potential to be developed into a novel HIV-1 entry/fusion inhibitor (**Table 2**).

**Table 2:** *In vitro* cell viability of NSPD-12m.

Cells	CC_50_ (µM)	CC_90_ (µM)
U87-CD4-CCR5	84.70 ± 14.54	101.71 ± 18.30
U87-CD4-CXCR4	101.36 ± 0.48	122.54 ± 0.59
TZM-bl	78.99 ± 11.37	118.28 ± 17.97
MT-2	67.12 ± 10.96	123.60 ± 70.16
CHO-WT	59.98 ± 13.16	74.60 ± 13.81

### NSPD-12m Inhibited the Single-Cycle Infection by HIV-1 Env Pseudotyped Viruses

**Figure 2:**
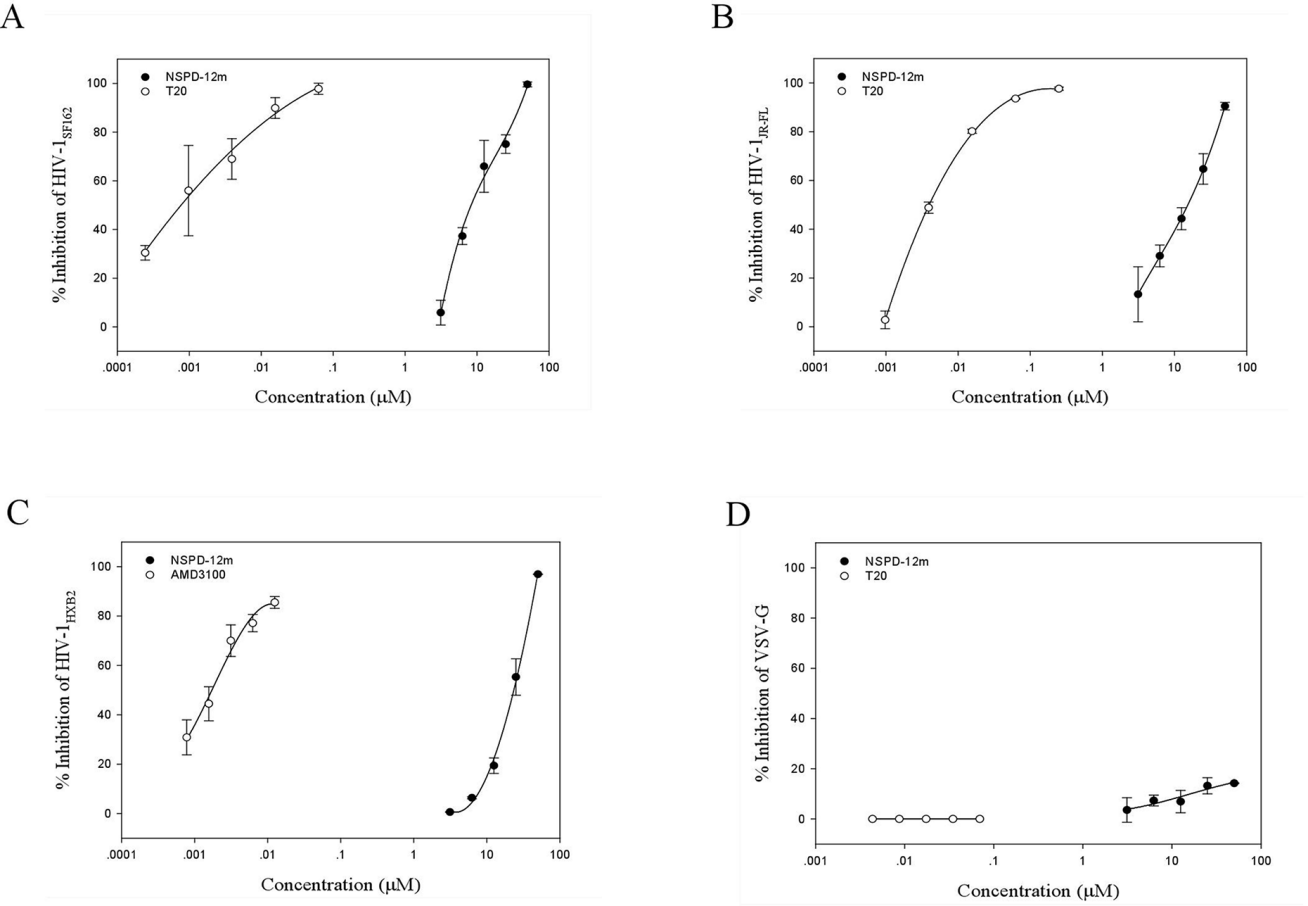
The inhibitory activity of NSPD-12m on infection by HIV-1_SF162_ **(A)**, HIV-1_JR-FL_ **(B)**, HIV-1_HXB2_ **(C)**, and VSV-G **(D)** pseudotyped virus. The samples were tested in triplicate, and the data were presented in mean ± SD.

To clarify the possible mechanism of action of NSPD-12m, we determined the antiviral activities of NSPD-12m against single-cycle infection by different HIV-1 Env pseudotyped viruses. As shown in **Figures 2A–C**, NSPD-12m had potent inhibitory effects on infection by all tested HIV-1 Env pseudotyped viruses, including HIV-1_SF162_ (R5 strain), HIV-1_JR-FL_ (R5 strain), and HIV-1_HXB2_ (X4 strain), with EC_50_ values of approximately 19.65, 13.95, and 23.09 µM, respectively. The results showed that NSPD-12m had similar inhibitory activities against both the HIV-1 X4 and R5 strains, suggesting that NSPD-12m displayed broad anti-HIV activities and had no significant coreceptor tropism. The positive control drugs, T20 and AMD3100 (a CXCR4 coreceptor inhibitor with anti-HIV-1 activity), had strong inhibitory activity against the R5 and X4 HIV-1 strains (**Figures 2A–C**). Here, a VSV-G pseudotyped virus expressing the VSV-G envelope was used as a negative control to evaluate the specificity of NSPD-12m for the HIV-1 envelope. The results showed that VSV-G pseudovirus infection could not be strongly inhibited by NSPD-12m at the concentration below 50 µM (**Figure 2D**), which indicated that NSPD-12m might be an HIV-1 entry/fusion inhibitor that targets HIV-1 envelope proteins specifically.

### NSPD-12m Inhibited HIV-1 Entry by Blocking HIV-1 Env-mediated Cell–Cell Fusion

**Figure 3:**
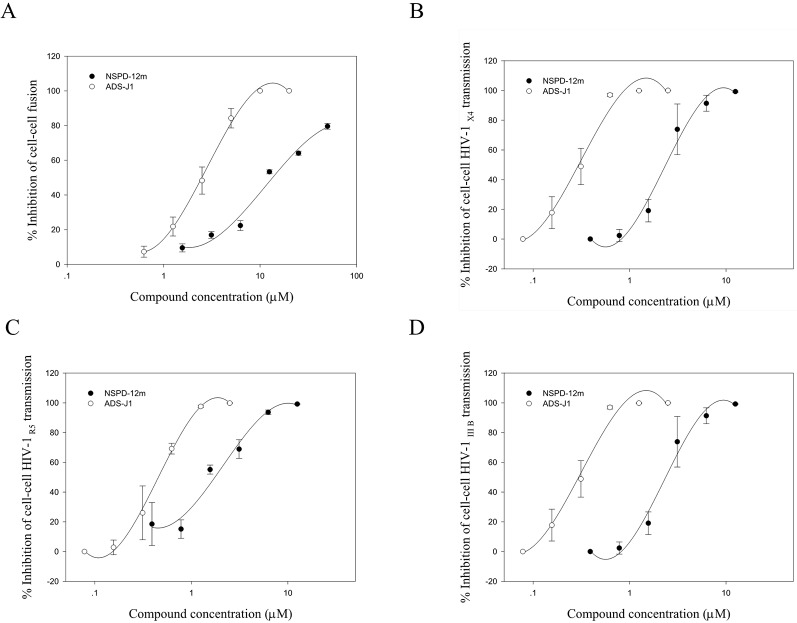
The function of NSPD-12m as a viral entry inhibitor confirmed by HIV-1-mediated cell-cell fusion **(A)**, cell–cell HIV-1 X4 transmission **(B)**, cell–cell HIV-1 R5 transmission **(C)**, and cell–cell HIV-1 IIIB transmission **(D)** assay. Each sample on was tested in triplicate and data were presented in means ± SD.

Cell–cell fusion can mimic the critical step of viral Env-mediated membrane fusion and HIV entry into a target cell during the viral life cycle *in vitro*. First, cell–cell fusion was assessed by a syncytium-formation assay using a noninfectious system as previously described. Here, CHO-WT cells expressing HIV-1 Env were used as the effector cells, and MT-2 cells expressing the CD4 receptor and the CXCR4 coreceptor were used as the target cells. The results showed that NSPD-12m clearly inhibited the syncytium formation in a dose-dependent manner, with an EC_50_ value of 14.44 ± 1.24 µM (**Figure 3A**). In addition, we detected the inhibition of NSPD-12m on the transmission of cell-associated HIV-1 virus from MT-2 cells to CEMx174 5.25 M7 cells, which express CD4, CXCR4, and CCR5. As shown in **Figures 3B–D**, NSPD-12m blocked the cell–cell HIV-1 X4, R5, and IIIB transmission between the two kinds of virus target cells, with EC_50_ values of 3.64 ± 0.34, 1.42 ± 0.30, and 1.99 ± 0.08 µM, respectively.

The HIV entry/fusion inhibitors ADS-J1 exhibited a similar dose-dependent curve (**Figure 3**). Those results suggested that NSPD-12m might inhibit HIV entry/fusion by interfering with HIV-1 Env-mediated membrane fusion specifically.

### NSPD-12m Inhibited HIV-1 Gp41 6-HB Formation

**Figure 4:**
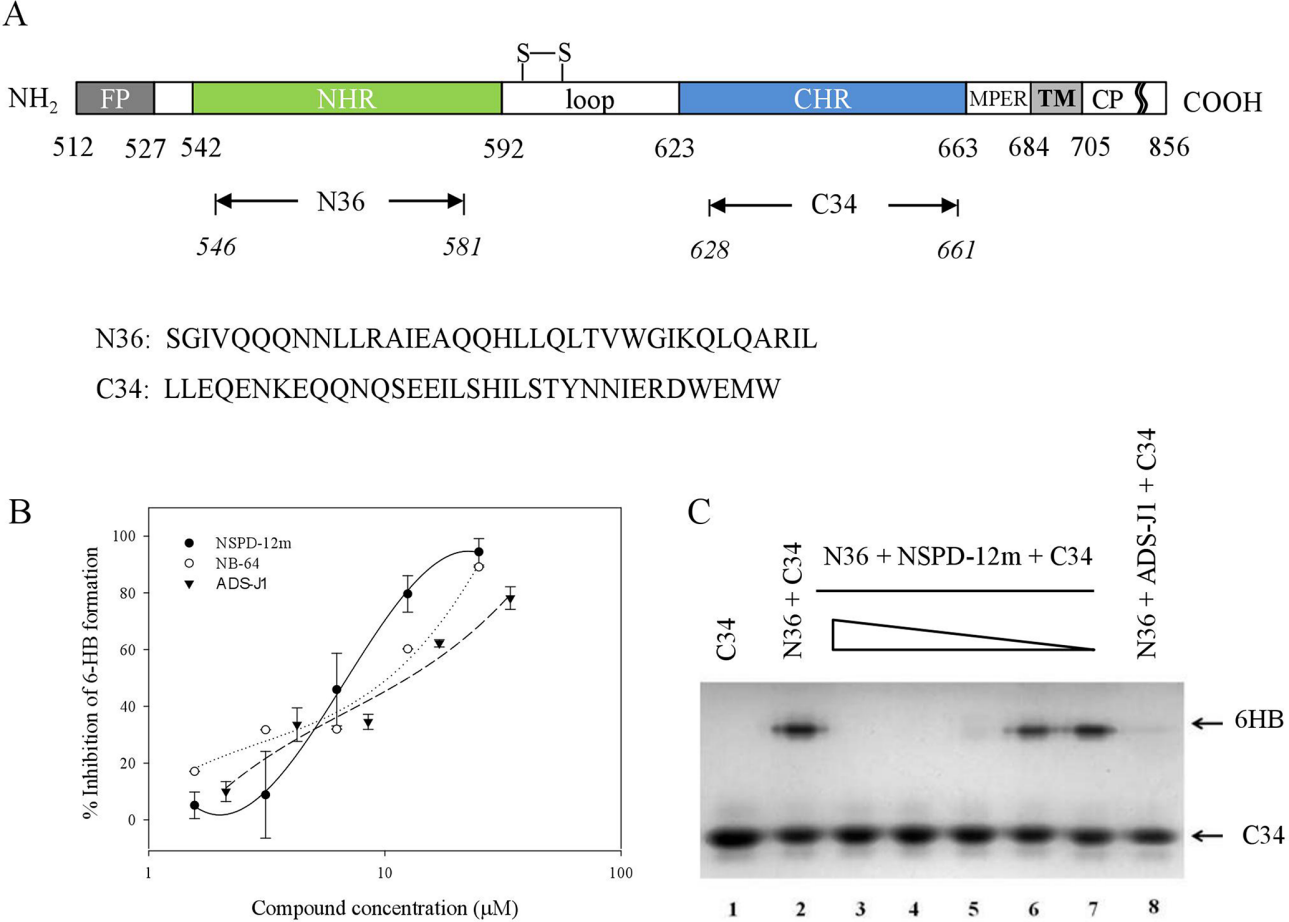
NSPD-12m inhibited the interaction between the NHR and CHR peptides to form the gp41 six-helix bundle formation. **(A)** The schematic view of the gp41 functional regions. **(B)** 6-HB formation was measured by a sandwich ELISA with mAb NC-1, NB-64 is a positive control. **(C)** 6-HB formation was measured by N-PAGE. Lane 1 is the peptide C34 and lane 2 is the mixture of peptide N36 and C34. The peptide N36 was incubated with NSPD-12m at graded concentrations (400, 200, 100, 50 and 25 mM for lanes 3 to 7, respectively) at 37°C for 30 min before addition of the peptide C34. Lane 8 is a positive control by ADS-J1 at 1 mM.

The stable HIV-1 gp41 6-HB formed by its N- and C-terminal heptad repeat sequences (NHR and CHR) (**Figure 4A**) is a key structure during HIV-1 fusion with target cells. Our groups have established several screening model systems to evaluate the effects of compounds on the formation of the 6-HB between the N36 and C34 peptides at the same molar concentration, including a capture sandwich ELISA and N-PAGE assay as described previously (Liu et al., 2005). The N- and C-peptides can mimic gp41 core formation *in vitro*, and the 6-HB structure can be identified using a conformation-specific mAb, NC-1, as reported earlier (Jiang et al., 1998). The results showed that NSPD-12m could block gp41 6-HB formation in a dose-dependent manner, with an EC_50_ of 23.05 ± 1.44 µM measured by ELISA (**Figure 4B**). NB-64 and ADS-J1 were used as positive controls. The inhibitory activity of NSPD-12m against 6-HB formation was further verified by a convenient N-PAGE assay. As shown in **Figure 4C**, the C34 peptide alone showed a clear band in the native gel at the lower position (lane 1). When the C34 peptide was mixed with the N36 peptide, a specific and visible band at the upper position corresponding to the 6-HB structures was revealed on the gel (lane 2). After incubating the different concentrations of NSPD-12m (400, 200, 100, 50, and 25 μM) (lanes 3–7) with N36 before the addition of C34 peptides at 37°C, the intensities of the specific bands at the upper position were decreased, while those of the lower bands were increased correspondingly in a dose-dependent manner (**Figure 4C**). ADS-J1 at 1 mM (lane 8), an HIV-1 entry inhibitor that interacts with the gp41 pocket region and blocks the formation of the fusion-active gp41 core, was used as a positive control. These results certified that gp41 6-HB formation could be inhibited by NSPD-12m significantly.

### NSPD-12m Interfered With the α-Helical Conformation Formed by N- and C-Peptides

**Figure 5:**
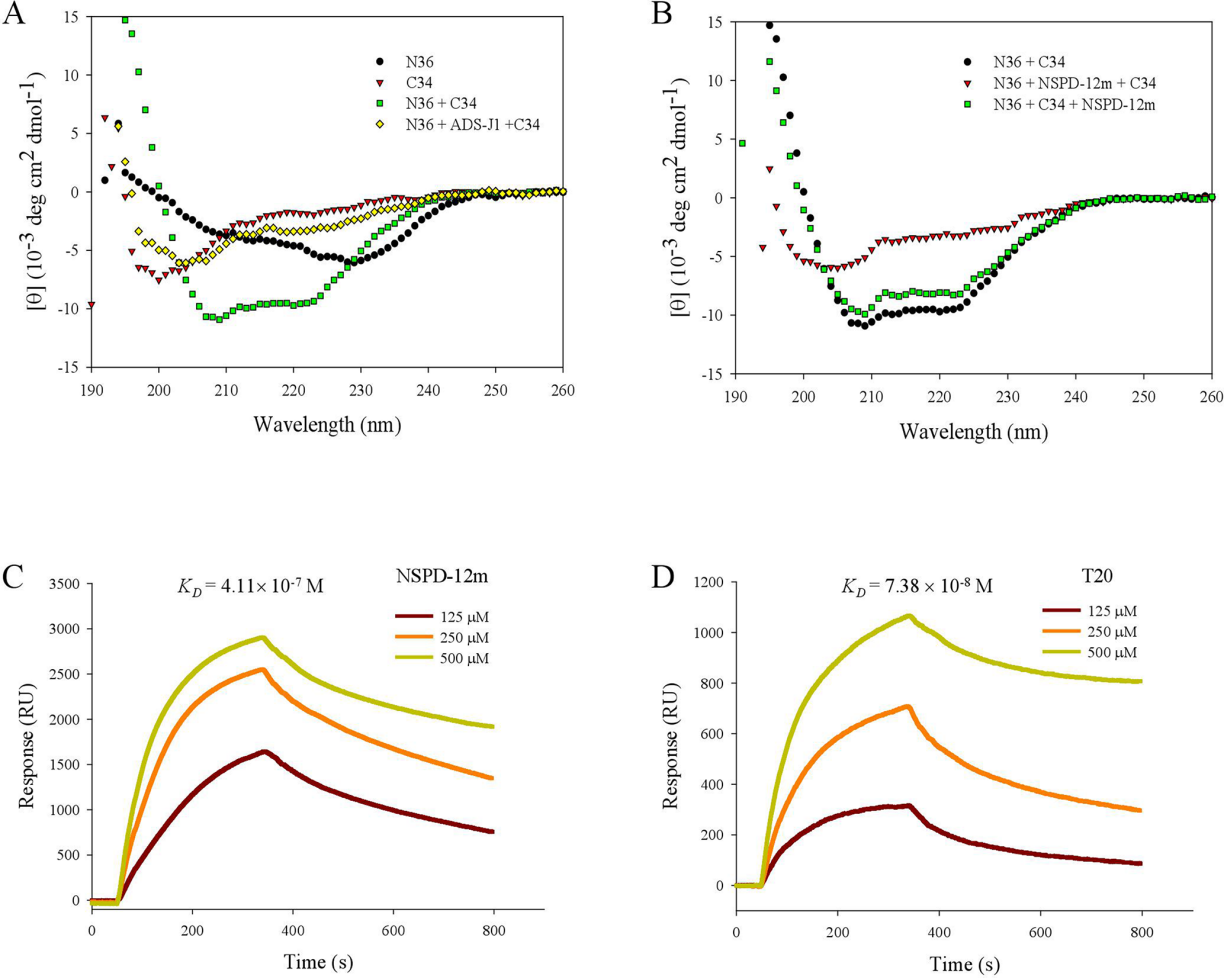
NSPD-12m interfered with the α-helical conformation formed by NHR and CHR peptides as detected by CD spectroscopy and SPR analysis. **(A)** N36 was incubated with ADS-J1 or PBS before the addition of C34 by CD. **(B)** N36 was incubated with NSPD-12m before or after the addition of C34 by CD. Dose-dependent binding of N36 with NSPD-12 m **(C)** or T20 **(D)** by SPR assay.

Furthermore, we investigated the conformational changes of the mixture of N36 and C34 peptides in the presence and absence of NSPD-12m by CD spectroscopy. Previous studies demonstrated that the CD spectra of the isolated N36 peptides or isolated C34 peptides showed random coil structures in aqueous solution (Lu et al., 1995), while the mixture of N36 and C34 peptides showed a typical α-helical coiled-coil secondary structure conformation. As shown in **Figure 5A**, the CD spectra of the mixture of N36 and C34 peptides showed a typical saddle-shaped negative peak and a significantly increased molar ellipticity (㪌θ) at 222 nm. However, the α-helical content of the 6-HB of the mixture of N36 and C34 peptides in the presence of 20-µM NSPD-12m was clearly reduced when the compound was preincubated with the N36 peptide before the addition of the C34 peptide. This result indicated that NSPD-12m interfered with the development of the α-helical conformation between N36 and C34 peptides (**Figure 5B**). Interestingly, NSPD-12m had no significant effects on the development of the α-helical coiled-coil conformation of the 6-HB by N36 and C34 peptides (**Figure 5B**). Here, a small-molecule HIV-1 entry inhibitor that blocks gp41 6-HB formation, ADS-J1 (40 µM), was used as a positive control (**Figure 5A**).

The SPR assay was also utilized to elucidate the interaction of NSPD-12m with the N36 peptide. In this experiment, NSPD-12m (10 mM) was immobilized on a sensor chip, and N36 at graded concentrations was injected onto the immobilized surface. As shown in**** **Figure 5C**, NSPD-12m could strongly bind to N36 with a binding affinity (*K_D_*) of 4.11 × 10^−7^ M. The positive control, T20, bound to N36 with a *K_D_* of 7.38 × 10^−8^ M (**Figure 5D**). These results indicated that NSPD-12m could inhibit the formation of the α-helical complex between the NHR and CHR peptides by binding to the NHR peptide.

### Lys574 was a Key Residue in the Inhibitory Activity of NSPD-12m on HIV-1 Infection

**Figure 6:**
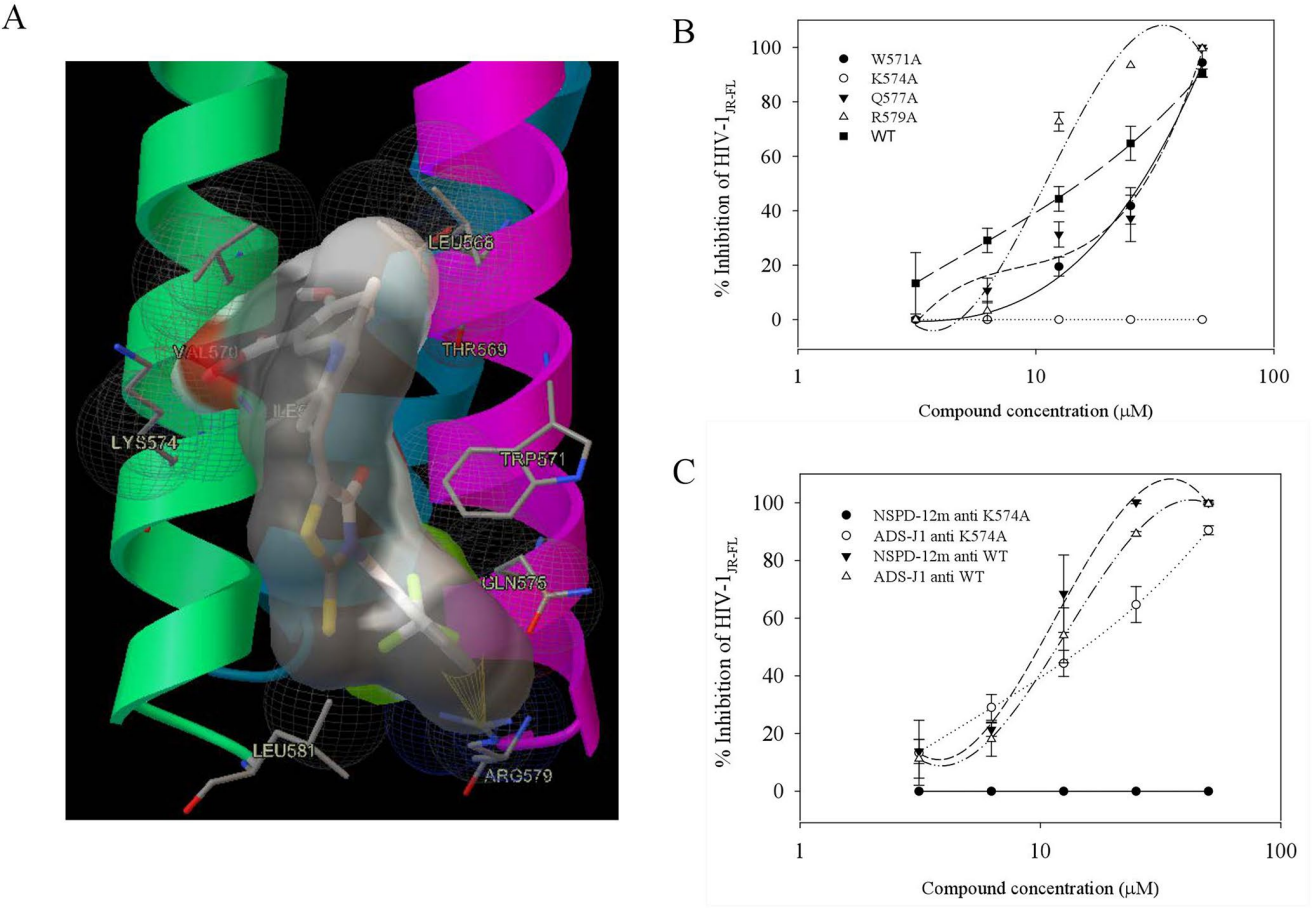
Site-directed mutagenesis analysis. **(A)** NSPD-12m and its docking conformation inside the hydrophobic pocket of HIV-1 gp41. **(B)** Inhibitory activity of NSPD-12m on infection of the mutant HIV-1 W571A, K574A, Q577A, and R579A pseudoviruses. **(C)** Inhibitory activity of ADS-J1 on infection of the mutant HIV-1 K574A pseudoviruses. The samples were tested in triplicate, and the data are presented in mean ± SD.

Our group had previously reported that a highly conserved, positively charged residue, Lys574, in the hydrophobic cavity of the HIV-1 gp41 coiled-coil domain might be a key target for the inhibitory activities of a series of small-molecule HIV-1 entry/fusion inhibitors derived from NB-64 (He et al., 2007). Here, the possible binding site(s) of NSPD-12m to the gp41 6-HB coiled-coil domain was also evaluated using molecular docking analysis. The macromolecule for docking analysis was downloaded from RCSB database (PDBID: 1aik). The PDB structure 1AIK contained three models. Each model contain two chains (N36 and C34), which together composed the six-helix structure. To expose the hydrophobic pocket, one of the C34 chains was deleted after preparation of the receptor, as previously described (He et al., 2013). The residues K574, Arg579, and Gln577 were selected as flexible residues. As shown in **Figure 6**, our docking studies found that NSPD-12m partially occupied the gp41 binding pocket by binding to several key residues on the surface of the “pocket” to form a “salt bridge” through electrostatic interactions. Comparison of the chemical structures showed that relative to NB-64 and other related derivatives targeting HIV-1 gp41, NSPD-12m occupied more modifiable chemical space in the hydrophobic pocket of gp41 due to the presence of a rhodanine ring (C-ring) and a phenyl ring (D-ring) in its structure (**Figure 6A**). The double bond linker between the B-ring and the C-ring increases the molecular flexibility of NSPD-12m. This flexibility makes it easier to adjust the binding conformation around the key residue (K574), produce a salt bridge between the thioxo group and the side chain of K574, and form the cation-*p* interaction between the phenyl ring and K574. These changes indicated that a flexible linker between the B and C rings might be important for enhancing the biological activities of those small-molecule compounds. In addition, the D-ring partly filled a hydrophobic cavity formed by the residues W571, Q577, and R579 of the NHR and the fluorine combined with R579 to provide more interactions with the target cavity. Additionally, the compounds derived from NB-64 may also fit the hydrophobic cavity and interact with other key amino acids, such as W571, Q577, and R579, which are located in the N-helix coiled-coil domain of HIV-1 gp41 (He et al., 2011). Thus, we determined which part of those residues in the hydrophobic cavity of HIV-1 gp41 coiled-coil domain is critical for the antiviral activity of NSPD-12m. Here, we replaced the non-conserved residues W571, K574, Q577, and R579 with alanine (Ala, A) (He et al., 2007) by site-directed mutagenesis analysis. After concentrating those mutant pseudoviruses by PEG-it^™^ Virus Precipitation Solution, the inhibitory activities of NSPD-12m against infection by a panel of HIV-1_JR-FL_ Env mutants (K574A, W571A, Q577A, and R579A) were evaluated. As shown in **Figure 6B**, NSPD-12m retained the inhibitory activity against infection by three kinds of HIV-1_JR-FL_ Env mutants (W571A, Q577A, and R579A), with similar EC_50_ values to those of the wild-type pseudovirus. Strikingly, the nonconservative substitution of K574 with alanine (A) resulted in significant loss of NSPD-12m-mediated anti-HIV-1 activity (**Figure 6B**). ADS-J1 exhibited similar dose-dependent curves for inhibition of both wild-type and K574A mutant pseudovirus (**Figure 6C**). These results suggested that K574 might play an important role in mediating the antiviral activity of NSPD-12m.

## Discussion

There has been increased interest in developing HIV entry/fusion inhibitors as new antiviral drugs that can work before the virus enters the host cell and can be used for prophylaxis. Most HIV-1 fusion/entry inhibitors that are currently in clinical trials or preclinical studies are peptides derived from the HIV-1 gp41 transmembrane glycoprotein (Henrich and Kuritzkes, 2013). However, the pharmacokinetic drawbacks of these peptides including T20, such as poor oral bioavailability, short-half life, and rapid renal clearance *in vivo*, limit the range of clinical applications (Lu et al., 2016). In addition, these peptides cause various adverse reactions, such as injection site reactions, and they are characterized by high molecular weight, high cost of production, and HIV-1 resistance. Considering the low price, good efficacy, safety, and ease of use of anti-HIV drugs, small-molecule compounds are attracting increasing attention. In the previous study, our group first reported that NB-64, which is an N-substituted pyrrole derivative and contains a COOHC group, displayed anti-HIV activities targeting the HIV-1 entry step (Jiang et al., 2004). To develop new HIV-1 entry/fusion inhibitors with improved antiviral activities and resistance profiles, we designed a small-molecule compound NSPD-12m derived from NB-64. In this study, we evaluated the antiviral activity of NSPD-12m against HIV-1 isolates with different genotypes and biotypes. The results showed that NSPD-12m exhibited potent inhibitory activities against all five HIV-1 isolates (**Table 1**). Furthermore, NSPD-12m was shown to be effective against T20-resistant HIV-1 strains (**Table 1**), indicating that it has the potential to block HIV-1 strains that are resistant to the currently used HIV entry/fusion inhibitor.

We further attempted to determine which step of the HIV-1 entry/fusion process is affected by NSPD-12m and to clarify its detailed mechanism of action in blocking HIV entry/fusion. Membrane fusion between the virus and cellular membranes is the first key step during the life cycle of HIV-1 (Melikyan, 2008; Harrison, 2015), which is mediated by the HIV-1 gp41. Here, HIV-1 Env-mediated cell–cell fusion and pseudovirus-mediated virus–cell fusion were used to detect the inhibitory action of NSPD-12m during the viral entry process. The results showed that NSPD-12m has specificity and a broad spectrum of antiviral activities by blocking the virus–cell and cell–cell fusion with EC_50_ values at low µM levels, implying that NSPD-12m might affect HIV-1 entry/fusion step (**Figures 2** and **3**).

As described previously, the HIV-1 gp41 subunit achieves membrane fusion by undergoing drastic conformational changes to form a six-helix bundle (6-HB) composed of NHR and CHR. Generally, the inhibition of 6-HB formation can cause the abortion of membrane fusion. A series of biological and physical methods have been conducted to verify the effect on 6-HB formation. However, the exact mechanism of the potential association of 6-HB with the fusing lipid bilayers remains elusive (Yamamoto et al., 2019). Here, sandwich ELISA, N-PAGE, CD spectroscopy, and SPR analyses were used to detect the inhibition of NSPD-12 on the formation of 6-HB. As shown in **Figures 4** and **5**, NSPD-12m could interfere with the interaction of NHR and CHR on gp41 and subsequently block the development of the 6-HB structure. Here, we used ADS-J1 as a positive control. ADS-J1 is a potential HIV entry inhibitor that inhibits gp41 6-HB formation (Debnath et al., 1999; Jiang et al., 1999; Jiang and Debnath, 2000). In addition, fluorescence-linked immunosorbent assay (FLISA)- and fluorescent resonance energy transfer (FRET)-based high-throughput screening (HTS) assays are more reliable for screening small-molecule HIV entry/fusion inhibitors by blocking 6-HB formation (Liu et al., 2003; Cai and Gochin, 2007). Extensive studies have examined the functional importance of each amino acid in the CHR by alanine scanning mutagenesis and confirmed that gp41 is a vulnerable target for therapeutic and prophylactic intervention (Diaz-Aguilar et al., 2013). Some small-molecule compounds that imitate peptides have also shown promise against gp41, such as O2N-[Ala]-Nap-OH, which was screened by a competitive inhibition fluorescence intensity assay (Gochin et al., 2013), and 14g, which was developed by structure-based design (Zhou et al., 2011).

It is worth noting that the hydrophobic pocket in grooves on the surface of the NHR-trimer is an attractive target for the development of small-molecule HIV fusion inhibitors (Chan et al., 1997; Chan et al., 1998). ADS-J1 was the first such molecule discovered by using a virtual screening program (Debnath et al., 1999), followed by NB-2 and NB-64 (Jiang et al., 2004). However, both NB-2 and NB-64 are considered too small to occupy the entire hydrophobic pocket, which can accommodate a 600-Da molecule. Molecular docking analysis indicated that a positively charged residue (K574) around the pocket in the NHR of HIV-1 gp41 might be a key target for the inhibitory activities of the small-molecule HIV-1 entry/fusion inhibitors derived from NB-64 (He et al., 2013). The residues W571, Q577, and R579 around the pocket might also be important for stability of the gp41 core structure (He et al., 2011). Some studies have also revealed that the hydrophobic groups (phenyl, naphthalene) of ADS-J1 might interact with the hydrophobic residues (Leu568, Val570, Trp571) in the hydrophobic cavity and that one of the sulfonic acid groups (SO3H) is in close proximity to Lys574, enabling ionic interactions (Debnath et al., 1999). Here, we demonstrated that NSPD-12m might fill the linear binding cavity and interact with the key residue, K574, using molecular docking analysis (**Figure 6A**). The pseudoviruses results showed that the K574A mutation rendered the viruses highly resistant to NSPD-12m, suggesting that K574 is the critical residue involved in the binding of NSPD-12m, which is consistent with the prediction from the computer-aided docking analysis of NB-64 (**Figure 6B**). According to the molecule docking analysis, the D-ring, particularly the fluorine, combined with W571, Q577, and R579. However, the pseudoviruses bearing a W571A, Q577A, or R579A mutation were not resistant to NSPD-12m, suggesting that the o-substituted phenyl moiety on the D-ring would not help increase the inhibitory 6-HB formation capability. In the present study, we found that ADS-J1 exerted inhibitory activity against the K574A mutant pseudovirus (**Figure 6C**).

Other studies have reported that a single polypeptide, denoted 5-Helix, displays potent (nanomolar) inhibitory activity and may be developed into an HIV-1 entry/fusion inhibitor targeting the gp41 CHR region (Root et al., 2001). The major difference between 5-Helix and 6-HB is that 5-Helix contains five of six α-helical coils and exposes one of the three grooves to attract a C-helix to fill in the gap and prevent 6-HB formation. Therefore, 5-Helix can also be used as a good target for screening HIV-1 fusion/entry inhibitors due to its exposed groove and hydrophobic pocket (Root and Steger, 2004). 5M038 and 5M041 were discovered through the 5-Helix-based HTS assay and inhibit HIV-1 Env-mediated cell–cell fusion at low μΜ concentrations (Frey et al., 2006). However, 5-Helix might not be able to be developed as a therapeutic drug due to its high molecular weight and may not be suitable for screening lead compounds with relatively low binding affinity to the pocket due to its high binding affinity (Lu et al., 2016).

Many computational studies have been reported on HIV-1 entry inhibitors. These studies have contributed useful information about the interactions between HIV-1 entry-related targets and their inhibitors, elucidating the most critical residues in these targets and providing insights into their mechanisms of action. Furthermore, studies have not been limited to only the hydrophobic pocket of gp41. Chu and Gochin reported a novel series of fragment binders to a relatively shallow subsite close to the pocket, which may facilitate binding as a hydrophobic pocket that tethers binders (Chu and Gochin, 2013). Small molecules may improve the inhibit potency by tethering binders of each pocket together. More recently, some compounds had been identified by computational screening based on their predicted binding to theoretical pockets on the inner groove of two N helices, thereby preventing N-helical trimer formation (Allen et al., 2015). This information has aided in identifying potent inhibitors, offering hope for discovering more effective HIV-1 entry inhibitors in the future.

Generally, safety is one of the most critical factors of small-molecule antiviral inhibitors that are considered drug candidates. A major factor preventing the clinical use of ADS-J1 is its low selectivity index (SI), as its long-term use may be potentially harmful. Higher molecular weight (1,177 Da) reduced its medicinal properties, while the azo bonds contained by ADS-J1 as a dye enhance its toxic effects. These characteristics limit the use of ADS-J1 as an HIV-1 fusion inhibitor. Although NSPD-12m had minimal effects on *in vitro* cell viability at the concentrations studied here (**Table 2**), the cytotoxicity of this compound should not to be ignored. Further structural modifications are needed to reduce cytotoxicity and increase anti-HIV-1 potency.

In conclusion, the present study demonstrated that NSPD-12m, which was developed from NB-64, showed significant inhibitory activity against HIV entry, probably by binding to the positively charged K574 residue in the pocket-forming region of the NHR. Although the anti-HIV-1 potency of NSPD-12m is not high enough for NSPD-12m to be considered a drug candidate, NSPD-12m more strongly disrupted 6-HB formation than did NB-64. Thus, NSPD-12m may be used as a lead for designing more potent small-molecule HIV-1 fusion inhibitors, and this study provides a new starting point for further structural modifications.

## Ethics Statement

The research protocols for this study were approved by the Ethical Committee of Nanfang Hospital and were performed in accordance with relevant guidelines and regulations.

## Author Contributions

JQ and LL: Designed the research and wrote the manuscript; JQ, TL, FY, and YT: Performed the experiments and analyzed data; XH, LX, and SJ: Contributed reagents/materials/analysis tools; JW and SL: Critically reviewed the study data and the manuscript. All authors approved the content of the manuscript.

## Funding

This study was supported by the Natural Science Foundation of China (81673481 to LL, 81503116 to JQ, and 81501735 to FY) and by the Natural Science Foundation of Guangdong Province (2014A030310044 to JQ).

## Conflict of Interest Statement

The authors declare that the research was conducted in the absence of any commercial or financial relationships that could be construed as a potential conflict of interest.
